# An Evaluation of the Reliability of the Results Obtained by the LBET, QSDFT, BET, and DR Methods for the Analysis of the Porous Structure of Activated Carbons

**DOI:** 10.3390/ma13183929

**Published:** 2020-09-05

**Authors:** Mirosław Kwiatkowski, Elżbieta Broniek

**Affiliations:** 1 Faculty of Energy and Fuels, AGH University of Science and Technology, al. A. Mickiewicza 30, 30-059 Krakow, Poland; 2 Faculty of Chemistry, Wrocław University of Technology, Gdańska 7/9, 50-344 Wrocław, Poland; elzbieta.broniek@pwr.edu.pl

**Keywords:** activated carbons, porous structure, adsorption, activation, BET, DR, LBET, QSDFT

## Abstract

This paper presents the results of an analysis of the impact of the activator to the product of carbonized materials mass ratio on the porous structure of activated carbons prepared from mahogany, ebony, and hornbeam wood by carbonization and chemical activation with potassium hydroxide. The analyses were carried out on nitrogen adsorption isotherms using the Brunauer–Emmett–Teller (BET), Dubinin-Radushkevitch (DR), and Quenched Solid Density Functional Theory (QSDFT) methods, as well as the numerical clustering-based adsorption analysis (LBET) method. The activated carbons with the best adsorption properties and homogeneous surfaces were prepared at a mass ratio of *R* = 3. The analyses suggest the significant potential of producing adsorbents characterized by a large surface area and adsorptive capacity from raw materials such as mahogany, ebony, and hornbeam wood. The analyses in question also included an evaluation of the usability and reliability of the results obtained with the employed methods of structural analysis. Particular focus was placed on the limitations of adsorption models and on critically analyzing the output data. Our study shows the unique advantages of the LBET method compared to the other methods used. The LBET method allowed us, for example, to determine the degree of heterogeneity of the surface of the studied activated carbons and the shape of the clusters of adsorbate molecules formed in the pores of the studied material, as well as obtain information about the distribution of adsorption energy on the first adsorbed layer. This study also demonstrates the limitations of the methods used and the necessity to use LBET and QSDFT methods simultaneously for porous structural analysis. The simultaneous analysis of the adsorption isotherms via the LBET and the QSDFT methods makes it possible to choose the optimal preparation conditions while considering the properties of the original raw material. The analyses also suggest the complementary character of the employed methods and the scope of the useful and reliable information that can be obtained with these methods.

## 1. Introduction

New tasks related to protecting air and surface water, as well as implementing sustainable development principles, have resulted in an increased demand for environmentally friendly, cheap, and widely available adsorbents [1,2,3]. The most common adsorbents are activated carbons, which are composed of a carbon structure that contains small amounts of heteroatoms, such as oxygen and hydrogen, along with, depending on the type of raw material used, various mineral substances. The skeletal structure of activated carbons can be considered a mixture of graphite-like crystallites separated by disordered carbon consisting of complex aromatic-aliphatic forms and inorganic matter derived from the raw material. These graphite-like crystallites consist of several parallel flat graphite layers that are randomly oriented and interconnected.

Activated carbons have a very large specific surface area resulting from a significant proportion of micropores that constitute about 90–95% of the carbon’s total porosity. Most of the adsorption process takes place in the micropores, but larger pores, such as mesopores and macropores, also play a very important role in any adsorption process because they act as transport pores through which the adsorbate molecules reach the micropores [1,2,3].

The specific surface area of activated carbons is not always proportional to its adsorption capacity due to the pore diameter often being smaller than the diameter of the adsorbate molecule or the shape of the pores not allowing adsorbate molecules to penetrate into the micropores. Therefore, the proper selection of activated carbons for specific applications should also consider the size and shapes of the micropores, not just their specific surface areas and pore volumes.

The structure and most important properties of activated carbons depend on the type of raw material used to produce them, pre-treatment of the raw material, the conditions prevailing during the entire production process, and the final processing stages. Nearly any raw material containing carbon can be used for the production of activated carbons. However, in industrial practice, the factors that determine the choice of raw materials are the expected properties of the final product for specific applications and the economic aspects that affect the profitability of the production process on a commercial scale. The most commonly used raw materials for the production of activated carbons are hard coal, anthracite, lignite, peat, petroleum coke, pitch, and synthetic polymers. The demand for activated carbons is constantly increasing, and the production and regeneration of activated carbons made from the aforementioned raw materials are very expensive. Therefore, waste products from the agricultural, forestry, and food industries are being used increasingly more often for the production of activated carbons [4,5,6,7]. The production of activated carbons from biomass waste materials not only facilitates the efficient disposal of such materials but also transforms them into valuable materials used, for example, in environmental protection.

Activated carbons are produced from the process of physical activation preceded by carbonization or through the process of chemical activation [8,9,10,11,12,13,14,15,16,17]. However, these processes must be preceded by pre-processing of the precursor, including the stages of washing, drying, crushing, and milling, as well as sieving to the appropriate grain fraction. It is very important to wash the precursor with water, which is required for removing organic impurities, sand, dust, and other contaminants. After the activation process, these impurities become unwanted ash, the high content of which affects the adsorption capacity of activated carbons and negatively affects their mechanical strength. Water washing is effective, however, only for compounds loosely bound; the minerals that are strongly bound with the structure of the precursor require a more effective method that uses acids for their removal. A drying process is carried out after the washing stage, which facilitates subsequent mechanical processing of the raw material, i.e., crushing and milling.

Physical activation involves the process of carbonization in an inert atmosphere, followed by activation in an atmosphere of an oxidizing gas such as steam, carbon dioxide, and (optionally) a mixture of carbon dioxide CO_2_ and nitrogen N_2_ or air at a temperature of 1073.15 K to 1373.15 K [9,10]. The carbonization process consists of four main processes: the removal of structural moisture at a temperature below 473.15 K, the removal of volatiles at 443.15–543.15 K, decomposition of the organic substance of the raw material, and increasing the carbon content by removing the remaining volatile components in the final stage at a temperature above 623.15 K [9,10].

The specific surface area of the products of carbonization is usually less than 300 m^2^/g, which is insufficient for practical use in most industrial processes. Therefore, after the carbonization process, a chemical or physical activation process is required to develop the porosity of the material. The physicochemical nature of the raw material, the conditions of the carbonization process, the heating rate and time, and the final temperature affect the properties and porosity of the activated carbons. During activation, less organized carbon matter and some of the elemental carbon from the crystallites are removed from the space between the crystallites.

The activated carbons produced in the steam activation process has a larger specific surface area than the activated carbons obtained in the carbon dioxide activation process because water molecules are smaller in size and, therefore, can easily diffuse and react with the carbon substance. At the same temperature, the rate of steam activation is much higher and faster than activation using carbon dioxide. Therefore, activation using carbon dioxide is carried out at a temperature higher than that for steam activation [9,10]. However, the higher reactivity of steam activation results in the formation of a structure with a high proportion of mesopores, while CO_2_-activated carbons have a microporous structure. The disadvantages of this process also include its long process time and significant energy consumption.

The production of activated carbons by chemical activation consists of a one-step reaction of the raw material with an activating agent [11,12,13,14,15,16,17]. In the process of chemical activation, the raw material is first mixed or impregnated with a chemical compound—potassium hydroxide KOH, potassium carbonate K_2_CO_3_, phosphoric(V) acid H_3_PO_4_, sulphuric acid H_2_SO_4_, or zinc chloride ZnCl_2_. Then, this mixture is carbonized in an inert atmosphere. Chemical activation requires a washing step at the end of the whole process because the porosity formed in the carbon structure is blocked by chemical compounds that must be removed from the resulting activated carbon [11,12,13,14,15,16,17].

Individual chemical substances react differently with precursors and thus affect the activation process in different ways. Moreover, the technique used for mixing the activator and precursors may affect the properties of the activated carbon. In the dry process, a powdered activating agent is mixed directly with the raw material, after which the activation process is carried out. On the other hand, in the wet/impregnation process, the raw material, mainly biomass, is added to the water solution and activating agent. This mixture is then stirred continuously until the chemical is completely dissolved. The suspension is dried before the heat treatment process. Direct mixing with activators helps to increase the surface properties of carbon and adsorption compared to the wet impregnation method [12,13,14,15,16,17].

Zinc chloride ZnCl_2_ is a very effective activator that enables the creation of a developed pore structure while preventing the formation of tar and other undesirable products that can block previously developed pores [13,14,15]. Nevertheless, the disadvantages of using ZnCl_2_ as an activating agent include its corrosivity and toxicity, which prevent its use on an industrial scale. In addition, the activated carbon obtained in the process of ZnCl_2_ activation cannot be used in the pharmaceutical and food industries [13,14,15].

In recent years, potassium hydroxide KOH, which is a strong base that diffuses into the carbon layers and destroys the texture structure of a raw material creating microporosity in the carbon structure, is becoming increasingly popular as an activating agent [16,17]. Zinc chloride is the most effective activator in the processes for obtaining activated carbons, making it possible to prepared activated carbons with a very well-developed pore structure and a narrow range of microporosity without the participation of mesopores. Potassium hydroxide is more effective as an activator than its alternatives due to the formation of the free element K during the activation process and its intercalation in the structure of hexagonal planes built of carbon atoms [16,17]. The metallic potassium intercalated into the carbon material structures is removed in the subsequent HCl washing process, because of which voids are formed in the structure of the material. However, this compound is not completely evaporated during the activation process because its boiling point is 1600.15 K—much higher than the activation temperature. Therefore, it is very important to thoroughly wash the activated carbons obtained in the KOH activation process with water [16,17]. Due to its potential threat to the natural environment, potassium hydroxide is increasingly being replaced with potassium carbonate K_2_CO_3_.

Activated carbons are produced from different raw materials of organic origin including biomass waste. The choice of raw material for the production of activated carbons is determined by the ultimate designation of the activated carbons, as well as the availability and price of the raw material. The wood processing and carpentry industries, as well as the food processing industry, have a particularly strong potential for waste biomass, which can be successfully used for the production of activated carbons [18,19,20,21,22,23,24,25,26,27]. The arguments for using biomass waste in the production of activated carbons include biomass’s relatively low cost of production, the easy accessibility of its raw materials, and its renewability. From economic and ecological points of view, the adsorbents obtained from agricultural, forestry, and wood industry waste offer an alternative to adsorbents produced from hard coals and other precursors [28].

## 2. Materials and Methods

The present research considered the possibility to produce activated carbons from African wood, e.g., mahogany and ebony, as well as from European hardwood, e.g., hornbeam, via chemical activation with KOH. Here, particular focus is given to the influence of the mass ratio of the activator to the mass of the products of carbonized wood on the formation of the porous structure of the resulting activated carbons.

The activated carbons were obtained from mahogany (marked as MAC), ebony (EAC), and hornbeam wood (HAC), from which a 1–3.15 mm grain fraction was prepared and subjected to carbonization. The carbonization of the raw materials to the final temperature of 773.15 K was performed in a modified Fischer–Schröder (Wrocław University of Technology, Wrocław, Poland) retort in a nitrogen atmosphere at a heating rate of 5 K/min. The sample was maintained at the final temperature for an hour until the mass was stabilized, and then it was cooled in a nitrogen atmosphere until it reached room temperature.

The products of carbonized wood obtained from each of the raw materials were mixed with solid and powdered potassium hydroxide and triturated in a mortar to homogenize the mixture. For the purposes of this research, samples of activated carbons were obtained according to the mass ratio of the activator to the mass of the product of carbonized wood, i.e., the mass ratios *R* = 1, 2, and 3. After impregnation with potassium hydroxide, the samples were heated in a vertical tube furnace under a nitrogen flow (30 dm^3^/h) at a rate of 5 K/min until reaching the final activation temperature *T* = 1073.15 K. The activated carbon samples were subsequently kept at the final activation temperature for 1 h and then cooled to room temperature under a nitrogen flow. Next, the activated carbons were treated with 0.5 M HCl solution and washed with hot distilled water to wash out the chloride ions. The final stage of preparation of the samples involved drying the activated carbon at a temperature of 373.15 K to a constant weight.

Microporous activated carbons are highly complex both structurally and chemically, which renders a structural description of a particular material virtually unfeasible. As a result, a number of different mathematical models for both the porous structure of such materials and the adsorption processes on their surface have been developed, which significantly simplify the actual structures and mechanisms of the processes that take place on their surface. This issue becomes even more complex when analyzing the impact of the preparation conditions on the formation of the structure of activated carbons and the phenomena that occur during the production processes for such materials. Therefore this research involves a comparative analysis of the methods employed to describe the porous structure of adsorbents and the adsorption process on the surface of activated carbons (i.e., the Brunauer–Emmett–Teller (BET) [29] and the Dubinin-Radushkevitch (DR) [30] methods) based on models that offer the highest simplification of reality and, consequently, the least reliable ones. Moreover, the most recent methods for structural analysis were also applied using advanced mathematical and computer-based tools, considering in particular the surface heterogeneity of the analyzed material (namely, the numerical clustering based adsorption analysis (LBET) method [31,32,33,34,35] and the Quenched Solid Density Functional Theory (QSDFT) method [36,37,38,39,40]). Special attention was paid in the analyses to the limitations of the relevant theoretical models of the methods and critically analyzing the output data for their reliability and actual usability in research work.

Many methods have been devised to analyze porous structures based on adsorption isotherms. One of the most popular and commonly used methods is the BET method derived from the multi-layer adsorption theory by Brunauer, Emmet, and Teller, as well as the eponymous model developed from that theory [29]. According to the BET theory, the first adsorption layer is formed on active centers located on the adsorbent surface. However, it is assumed that the probability of occupying a given site is independent of the neighboring sites and the molecules that may already be present at the neighboring sites [29]. A criticism of the BET theory is its assumption that all adsorption sites on a solid surface are homogeneous in their energy. Moreover, the BET model does not consider interactions between the adsorbed molecules situated at neighboring sites.

The BET method is usually used to determine the specific surface area *S_BET_*; however, it is known for its limitations since the assumptions of the BET model do not consider the filling of the volume of the micropores. Hence, this method is not recommended for an analysis of microporous materials. Nevertheless, this method has been commonly used to determine the surface area of microporous activated carbons with little consideration for the meaningfulness of the results. Does it, therefore, make sense to employ this method in a study of the impacts of preparation conditions on the formation of the porous structures of carbonaceous materials? This method can, indeed, be used, provided that a comparison is made only between the values of the specific surface areas determined for the materials produced under different preparation conditions and bearing in mind the significant limitations of the method when used to analyze materials with a prevalent ratio of micropores to total porosity.

A popular equation used to describe adsorption on the surface of microporous carbonaceous materials is the DR equation. This equation was derived empirically based on the adsorption theory of the volume filling of micropores proposed by Dubinin and Radushkevich [30]. It can be employed to analyze the porous structure of activated carbons, provided that one bears in mind the various defects in the Dubinin–Radushkevich equation that affect the reliability of the calculation results. In particular, the adsorption isotherms of many microporous carbonaceous adsorbents usually cannot be fitted to the DR equation, and, in many cases, the fittings are unacceptable. Additionally, attention is paid to the impact of temperature on the obtained values of the volume of micropores caused by the presence of sub-micropores [23].

The BET and the DR equations are not guaranteed to provide a wide spectrum of reliable information on the microporous structure. This is because the prevalence of micropores in the total porosity of these materials affects the mechanism of the adsorption process. As a consequence, the description of structurally, geometrically, and energetically heterogeneous surfaces poses significant problems for identifying surface phenomena and their quantitative descriptions. Therefore, attempts are made to elaborate theoretical models of the specific characteristics of real-life microporous materials’ surfaces and to determine the correlation between heterogeneity parameters and the isotherms of the adsorption of vapors and gases. Considering the above factors, new more advanced methods have been devised to describe the adsorption process on heterogeneous surfaces. One such method is numerical clustering-based adsorption analysis (LBET) method, which is based on the unique LBET models that stem from the popular BET model. However, unlike the BET model, LBET models consider the surface heterogeneity, the possibility of adsorbate molecule cluster branching, and the geometrical and energy limitations of the formation of clusters of adsorbate molecules. The LBET method was supplemented with the advanced numerical fast multivariate procedure of adsorption system identification [31,32,33,34,35].

The LBET method, the theoretical foundations of the LBET models and their derivations, and the numerical fast multivariate procedure for adsorption system identification are described in detail in earlier publications. Here, we provide an overview of the most important information concerning the mathematical LBET models of adsorption on the homogeneous and heterogeneous surfaces of carbonaceous adsorbents that are implemented in this method and are indispensable for the correct interpretation of the results [31,32,33,34,35].

The LBET models were formulated assuming that each adsorbate molecule can occupy only one adsorption site. Moreover, it was assumed that each site available on that layer is equally probable to be matched with any adsorbate molecule, with no regard to the occupancy of other sites. Moreover, each cluster of adsorbate molecules contains a single molecule in the primary adsorption site, and the enlargement of a cluster does not impact the appearance and growth of other clusters [31,32,33,34,35].

Provided that each cluster of adsorbate molecules can occupy only a specified space and size, the general formula for the coverage ratio *θ_κn_* is expressed in the following form [31,32,33,34,35]:

(1) $$\theta_{\kappa n}=\frac{\Pi_{\kappa n}^{*}}{1+\Pi_{\kappa n}^{*}-\theta_{\kappa n+1}}\;\mathrm{for}\;n\;=\;k-1,\;\ldots 1,\;\Pi_{\kappa n}^{*}\overset{def}{=}\frac{\pi }{B_{\kappa n}{\left(1-\theta_{\kappa n+1}\right)}^{\beta_{\kappa n+1}-1}},\;B_{\kappa n}\overset{def}{=}\exp\left(\frac{Q_{\kappa n}}{RT}\right),$$

where index *κ* counts the *k*-th type clusters of adsorbate molecules for an identical energy profile across layers *n* = 1, …, *k*; *Q_κn_* is the molar energy contributed by placing an adsorbate molecule at the *n*-th layer of *κ*-th class clusters; *π* is the relative pressure of the adsorbate; *R* is the gas constant; *T* is the temperature; *k* is the maximum number of layers, i.e., the size of the molecule adsorbate cluster; and *β_κn_* is the pore shape factor.

For Equation (1) to be applicable when studying the adsorption systems of a random porous structure with *k* = 1, …, *K* ≈ ∞, it was assumed that for layers *n* = 2, …, *k*, *β_κn_* = *β*, and

(2) $$B_{C}\overset{def}{=}\exp\left(\frac{Q_{C}}{RT}\right),\;Q_{C}\overset{def}{=}U_{p}(1-2\cdot Z_{pp})-Z_{C}\cdot Q_{cp},\;$$

where *Q_cp_* is the molar adhesion energy in ideal adsorbent–adsorbate contacts, *U_p_* is the molar cohesion energy of the adsorbate in the bulk phase, and *Z_pp_*, *Z_C_* are the correcting factors.

Next, the first layer adsorption energy is expressed by the following equation [31,32,33,34,35]:

(3) $$Q_{A\kappa }=U_{p}-Z_{A\kappa }\cdot Q_{cp},\;Q_{A}\overset{def}{=}U_{p}-Z_{A}\cdot Q_{cp}=\underset{k=1}{\min}\left(Q_{A}{}_{\kappa }\right)$$

where *Z_Ak_* is the effective contact correction factor, and *ζ_Ak_* and *ζ_fk_* are the energy coefficients.

The number *m_hAk_* of the primary sites for *k*-th type clusters is

(4) $$m_{hA\kappa }=m_{hA}{\left(1-\alpha \right)}^{\alpha_{k-1}},\;\alpha \;\in \;\langle 0,\;1),$$

where *m_hA_* is the total number of primary sites, and *α* is the distribution parameter.

To increase calculation efficiency, we assumed that the coverage ratios *θ_κn_* are the same (*θ_κn_* = *θ*) and that the energetic parameters *B_Ak_* and *B_fk_* do not depend on *k*. Hence, the group of the LBET models is expressed using the following general form [31,32,33,34,35]:

(5) $$\begin{array}{c} \frac{m_{a}}{m_{hA}}=(1-\alpha )\left\{1-\frac{1}{\ln(B_{A}/B_{f1})}\cdot \ln\left(\frac{B_{A}+\pi }{B_{f1}+\pi }\right)\right\}+\\ +d\cdot \alpha (1-\alpha )\left(1+\beta \theta_{22}\right)\left\{1-\frac{1}{\ln(B_{A2}/B_{f2})}\ln\left(\frac{B_{A2}{\left(1-\theta_{22}\right)}^{\beta }+\pi }{B_{f2}{\left(1-\theta_{22}\right)}^{\beta }+\pi }\right)\right\}+\\ +\alpha^{d+1}\left[d+{\left(\beta \theta \right)}^{d}\left(1+\frac{\beta \theta }{1-\alpha \beta \theta }\right)\right]\left\{1-\frac{1}{\ln(B_{A2+d}/B_{f\theta })}\ln\left(\frac{B_{A2+d}{\left(1-\theta \right)}^{\beta }+\pi }{B_{f\theta }{\left(1-\theta \right)}^{\beta }+\pi }\right)\right\}, \end{array}$$

(6) $$B_{Ak}\overset{def}{=}B_{A}\cdot {\left(B_{C}\right)}^{Z_{A}(1-\zeta_{Ak})},\;B_{fk}\overset{def}{=}B_{A}\cdot {\left(B_{C}\right)}^{Z_{A}(1-\zeta_{fk})},$$

(7) $$B_{A}\overset{def}{=}\exp\left(Q_{A}/RT\right),\;B_{C}\overset{def}{=}\exp\left(Q_{C}/RT\right),$$

where *m_a_* is the total adsorption [mmol/g], *m_hA_* is the number of primary sites of the adsorbent pores (*m_a_* = *V_hA_*/*V_s_*), *V_hA_* is the volume of a space accessible to first-layer adsorption [cm^3^/g], *θ_kj_* is the coverage ratio of the *j*th layer of *k*th type clusters, *θ* is the coverage ratio of layers *n* > 1, *π* is the relative pressure, *α* and *β* are the geometrical parameters of the microporous structure (height and width of adsorbate clusters), and *B_Ak_*, *B_fk_* are the energy parameters for, respectively, the beginning and the end of the adsorption energy distribution. Further, *B_A_*, *B_C_* are the energy parameters for, respectively, the first and higher adsorption layers.

The LBET models have five parameters (*V_hA_* [cm^3^/g], *Q_A_* [J/mol], *α*, *β,* and *B_C_*), which can be adjusted by fitting Equation (5) to the adsorption isotherm with a chosen variant of the surface energy distribution function [31,32,33,34,35]. The energetic heterogeneity of the microporous adsorption systems significantly and negatively impacts the numerical conditioning of the system identification tasks. To solve this problem, a unique fast multivariate method for fitting the LBET class models to the adsorption isotherms was proposed, which is also employed to define the value of the surface heterogeneity indicator *h* and the shape of the distribution of adsorption energy on the first layer [31,32,33,34,35].

The advanced tools used to describe the porous structures of solids also include methods derived from density functional theory, which is a potential-based theory of adsorption, making it an advanced tool in mechanical statistics enhanced with computer-based calculation technology.

Density Functional Theory (DFT) aims to find the equilibrium condition of a liquid situated in the field of the external potential generated by the wall of an adsorbent pore, drawing on the density functional with the minimum value corresponding to the equilibrium profile of liquid density and allowing one to define the thermodynamic properties of an adsorption system [36]. Nevertheless, for molecular arrangements, the complete formula of the function is unknown, and its value can be determined in approximate terms only. Consequently, the most important challenge for DFT is finding approximations that afford a trustworthy and reliable description of the thermodynamic aspects of surface phenomena [36]. As a result, a range of methods have been defined within the framework of the formal approach behind the DFT. These methods differ from one another in their mathematical interpretations of the interactions between the adsorbate molecule and the walls of the pores in the material. The goal of these methods is to approach an equilibrium profile of density. Seaton et al. were the first to calculate the pore size distribution for both meso—and micropores on the basis of adsorption isotherms [37] by employing the Non-local Density Functional Theory (NLDFT) method and modelling pores as infinite slits between layers of graphite. Nonetheless, the NLDFT method is flawed because it does not consider (especially for microporous carbonaceous materials) the chemical and geometrical heterogeneity of pore walls. Instead, it assumes a pore model without a structure with a chemically and geometrically smooth surface [38]. Consequently, the NLDFT-based theoretical adsorption isotherms display numerous stages connected with layer transmission caused by the formation of the first and the subsequent adsorbed layers. This problem is revealed in the form of artificial gaps of about 1 and 2 nm in the determined pore size distributions [39].

Considering the above, a new method based on density functional theory was conceived: The Quenched Solid Density Functional Theory (QSDFT) method, which takes into account surface heterogeneity in one dimension and eliminates the artificial gaps in pore size distributions typical for calculations using NLDFT method [40,41,42,43,44]. The QSDFT model, like the NLDFT model, describes the adsorbent and the adsorbent density in one-dimension perpendicular to the pore wall. The QSDFT model also assumes that the carbon atom density decreases linearly to zero in the layer adsorbed on the surface.

**Figure 1:**
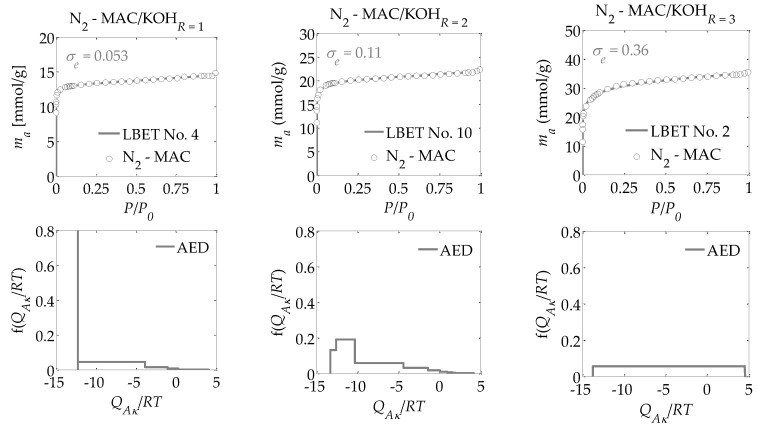
Nitrogen adsorption isotherms for the activated carbons MAC prepared from mahogany wood using KOH as an activator at a varying ratio *R* of the activator to the product of carbonized wood mass and the fast multivariate identification procedure results obtained using the LBET method, as well as the adsorption energy distributions in the first adsorption layer.

**Figure 2:**
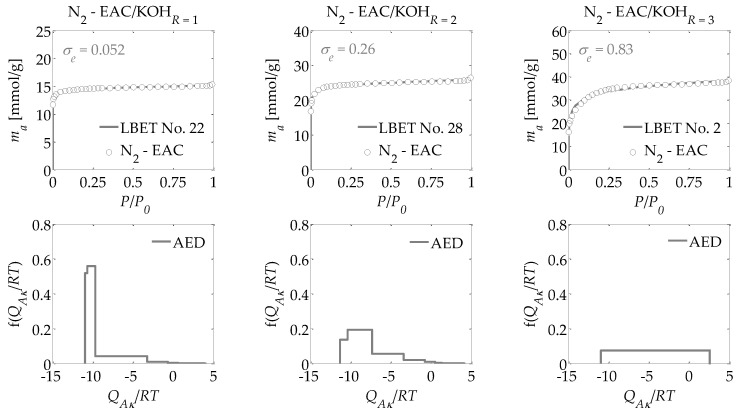
The same as Figure 1 but for the activated carbons EAC prepared from ebony wood.

**Figure 3:**
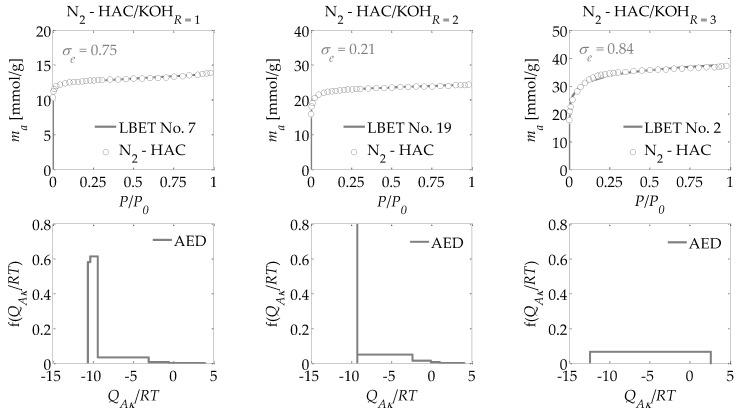
Same as Figure 1 but for the activated carbons HAC prepared from hornbeam wood.

**Figure 4:**
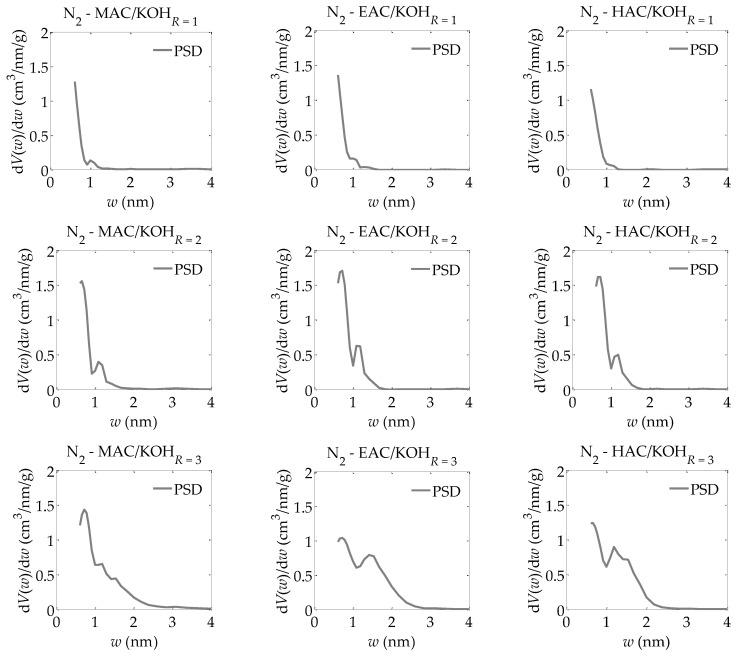
The pore size distributions obtained for all analyzed samples of activated carbons on the basis of nitrogen adsorption isotherms using the QSDFT method.

For the activated carbons obtained in the present research, nitrogen adsorption isotherms were determined at a temperature of 77.35 K. Based on these adsorption isotherms, the following were determined:

- The parameters of the capillary structure of activated carbons, i.e., the specific surface area *S_BET_* [29], the volume of micropores *V_DR_*, calculated from the DR equation [30], and *V_Total_*, i.e., the total pore volume, calculated from the maximum nitrogen adsorption at *P*/*P_0_* = 0.98 (Table 1);
- The parameters of the porous structure via the LBET method, i.e., the LBET class models with a fast multivariate numerical procedure for the identification of adsorption systems, including the volume of the first adsorbed layer *V_hA_*, the dimensionless energy parameter for the first layer *Q_A_*/*RT*, the dimensionless energy parameter for the higher layers *B_C_*, the effective contact correction factor *Z_A_*, the surface heterogeneity parameter *h*, the geometrical parameter of the porous structure determining the height of the adsorbate molecule clusters *α*, and the geometrical parameter of the porous structure determining the width of the adsorbate molecule clusters *β* (shown in Table 1) [31,32,33,34,35];
- The fitting error dispersion *σ_e_* and the identification reliability indices *w_id_* (shown in Table 1);
- The adsorption energy distributions on the first layer obtained by the LBET method based on the nitrogen adsorption isotherms for all the activated carbons prepared (Figure 1, Figure 2 and Figure 3);
- The pore size distributions obtained for the analyzed activated carbons using the QSDFT method [40,41,42,43,44] (shown in Figure 4).

## 3. Discussion of the Results

As gathered in Table 1, the results of the analyses carried out based on nitrogen adsorption isotherms obtained at 77.35 K demonstrate that with an increase in the mass of the activator to the mass of the products of carbonized woods, the values of the parameters *S_BET_*, *V_DR,_* and *V_Total_* increase gradually for all the analyzed activated carbons. The highest values for the volume of micropores *V_DR_* and total pore volume *V_Total_* were obtained for the activated carbons prepared from ebony, and the smallest ones were obtained for the materials prepared from mahogany. Activated carbons prepared from mahogany were also characterized by having the lowest values for specific surface area *S_BET_*. The highest value of the total surface area *S_BET_* was obtained for the activated carbons produced from hornbeam wood (HAC), while the highest value of the total pore volume was obtained for the activated carbons prepared from ebony (EAC). It should be emphasized, however, that these values do not differ significantly because the activated carbons were prepared from raw materials with similar characteristics, which was intentional.

It is not possible to use the obtained values of the parameters *S_BET_*, *V_DR,_* and *V_Total_* to recognize, with full reliability, either the porous structure of the activated carbons produced during the research or the processes taking place during the activation process with different ratios of the activating agent to the activated substance. Considering the above, the analysis of the materials during this research was also carried out using the LBET and the QSDFT methods, which consider, *inter alia*, the heterogeneity of the surface.

Drawing on the results obtained with the LBET method, it can be concluded that the prepared activated carbons are microporous, i.e., possessing a prevalence of micropores in their porous structures; an increase in the *R* ratio indicates an increase in the volume of the first adsorbed layer *V_hA_* and, consequently, in the proportion of micropores to total porosity. An increase in the *R* ratio is initially also parallel to the growth in the height of the adsorbate molecule clusters, as evidenced by the values of the *α* parameter. However, at *R* = 3, the *α* parameter decreases significantly, which is especially true for activated carbons produced from ebony and hornbeam wood. In turn, the *α* parameter analyzed in conjunction with the value of the *β* parameter that defines the width of the adsorbate molecule clusters indicates the adsorption of individual molecules of nitrogen on the surface of the analyzed materials.

The activated carbons prepared from the products of carbonized mahogany, ebony, and hornbeam wood at *R* = 1 are characterized by high and very high surface heterogeneity, respectively, which undermines the reliability of the results obtained with the BET and DR methods. Moreover, for the activated carbons prepared from products of carbonized mahogany and hornbeam wood, the *β* parameters that define the width of the adsorbate molecule clusters are 1.52 and 1.89, respectively, which conflicts with the assumptions of BET theory—namely, that only one molecule of the subsequent layer can be adsorbed into the molecule of a given adsorption layer. Thus, the *β* parameter should be 0. This, in turn, undermines the reliability of the determined *S_BET_* values.

For the samples of the activated carbons produced at *R* = 2, the *β* parameter values are equal to 1.00. However, for the samples prepared from mahogany and ebony, a very high degree of surface heterogeneity was found, which is defined by *h* parameter values of 7 and 9, respectively. The surface of the sample made from hornbeam wood was also heterogeneous, with *h* = 3. In light of the significantly higher proportion of micropores compared to the samples produced at *R* = 1, the results obtained using the BET and the DR methods were even less reliable. The activated carbons with the best adsorption and surface properties were obtained at a mass ratio of *R* = 3. For the activated carbons obtained at *R* = 3, the value for the degree of heterogeneity is the lowest at *h* = 1, yet these materials are characterized by the highest proportion of micropores in their total porosity. As such, these materials do not comply with the assumptions of BET theory.

Interesting conclusions can be drawn from the adsorption energy distribution results on the first adsorption layer of the adsorbent surface obtained using the LBET method. For the activated carbons prepared from products of carbonized mahogany, ebony, and hornbeam woods at a mass ratio of *R* = 1, the adsorption energy distribution points to the occurrence of a very narrow spectrum of sites with high adsorption energy and a smaller proportion of sites with a broad energy spectrum. On the other hand, the samples obtained at *R* = 2 from products of carbonized mahogany and ebony wood point to the occurrence of a broad spectrum of sites with different adsorption energies, though high-energy sites prevail. With the sample prepared from the product of carbonized hornbeam wood at *R* = 2, the adsorption energy distribution was characterized by a prevalent distribution of highly energetic sites. The activated carbons produced at *R* = 3 yielded an energy distribution shape that indicates a homogeneous distribution of the sites with different adsorption energies, which shows that these samples are characterized by the lowest energy heterogeneity of all the analyzed materials, which is also confirmed by the heterogeneity parameter *h* = 1.

Alongside the values of the structural parameters obtained in this study and the shape of adsorption energy distribution, there is the equally important issue of how reliable and precise this information can be. The LBET method is the only method with built-in tools to assess the reliability of results in the form of two indices: the fitting error dispersion *σ_e_* and the identification reliability index *w_id_*. The values of these indices for the individual adsorption systems are compiled into a table and demonstrate that the theoretical models fit the nitrogen adsorption isotherms very well. However, the values of the identification reliability indices *w_id_* that were obtained for the analyzed adsorption systems are merely average, which proves that the identifiability of the adsorption systems in question is far from ideal. Therefore, why does the mediocre identifiability of the adsorption systems coincide with a very good fit of the theoretical models to the analyzed adsorption isotherms? The reason probably lies in a feature of the analyzed structure that departs significantly from the assumptions of LBET models and can be solved only by using another method for the structural description—one that considers surface heterogeneity. This is why, as part of the present analyses, the pore size distributions were also determined for all the analyzed activated carbons obtained using the QSDFT method.

Based on the results of the calculations using the QSDFT method presented in Figure 4, very similar pore size distributions are observable for the activated carbons obtained from various materials, i.e., the products of carbonized mahogany, ebony, and hornbeam wood. These distributions have a bimodal structure of micropores, i.e., a dominant share of micropores in the first range from about 0.5 nm to 1 nm and in the second range from 1 nm to 2 nm, whereas with an increase in the ratio of the activator, i.e., potassium hydroxide, to the products of carbonized wood, the proportion of micropores increases successively in a range of 1 to 2 nm. The analysis carried out using the QSDFT method (especially the discovery of the bimodal structure of micropores) highlights the aforementioned problem of the average identifiability of adsorption systems when using the LBET method. The bimodal structure of the micropores was not accounted for in the LBET models, so its occurrence results in greater uncertainty in the reliability of the adsorption system identification.

It must be emphasized that the results obtained using the QSDFT method provided information that could not be gathered via the BET, DR, or LBET methods. More specifically, an increase in the ratio of the mass of the activating agent to the mass of the products of carbonized wood coincided with the development of a porous structure in the analyzed samples. However, this development occurred mostly in pores about 1 nm to about 3 nm in size, with minimum changes in a range from about 0.5 to 1 nm. Nevertheless, only with this information alongside the analytical results obtained using the LBET method can the mechanisms in place during the activation process be determined, thereby producing a more detailed picture of the structure of the pores in the analyzed materials. Considering the above, the QSDFT method should be used together with the LBET method to obtain reliable information on the structure of pores.

The results of this research also show that the outcome from using the BET and the DR methods is closely correlated with that when using the LBET and the QSDFT methods. Therefore, these methods can be considered useful for analyzing how preparation conditions impact the formation of the porous structures of carbonaceous adsorbents.

## 4. Conclusions

The results of the research presented in this article highlight the significant potential of producing activated carbons with a very high adsorption capacity and large specific surface area from mahogany, ebony, and hornbeam wood through activation with potassium hydroxide. The activated carbons with the best adsorption properties were prepared at a mass ratio of the activator to the products of carbonized wood equal to *R* = 3. In particular, the activated carbons prepared from the carbonized mahogany, ebony, and hornbeam wood in the process of carbonization and activation with potassium hydroxide at a mass ratio of *R* = 3 were characterized by the highest values of specific surface area, micropore volume, total pore volume, and volume of the first absorbed layer. Moreover, the activated carbons prepared at a mass ratio of *R* = 3 were characterized by a homogeneous surface, which is a very desirable property for determining the effective use of these materials in adsorption processes.

The activated carbons obtained as part of this research are, in fact, characterized by adsorption properties similar to those of the best carbonaceous adsorbents produced from homogeneous polymers. The above results demonstrate that the waste materials of the timber and carpentry industries can provide valuable raw materials for the production of high-quality activated carbons and their derivatives, i.e., monoliths of activated carbons that may be applied widely in many industries and in everyday life.

It should be emphasized that the present research yielded a broad spectrum of information and sheds new light on issues pertaining to assessing the effects of carbonaceous adsorbents’ preparation conditions on the parameters of porous structure development, which was also possible through application of the LBET and the QSDFT methods.

The LBET method—which allows one to determine the degree of heterogeneity on the surface and thus determines the possibility of using adsorbents in a given adsorption process—offered particular advantages in the conducted research. In addition, this method allowed us to determine the shapes of the clusters of adsorbate molecules formed in the pores of the material under study, as well as to obtain information about the distribution of adsorption energy on the first layer. The LBET method also makes it possible to determine the credibility and reliability of the results obtained, which is one of its unique features. However, as shown in this research, the use of the LBET method itself may, in some cases, be insufficient to precisely determine the effect of the preparation conditions on the formation of the porous structures of activated carbon. As demonstrated by the QSDFT method, the microporous structures of the analyzed types of activated carbons were bimodal, which explains the average identifiability of the adsorption systems using the LBET method, whose theoretical model does not consider such a case. However, the application of the QSDFT method alone or together with the BET and the DR methods is not itself sufficient to reliably analyze the influence of preparation conditions on the formation of a porous structure. Therefore, these complementary methods should be used together to analyze the porous structures of activated carbons to obtain complete and reliable information on the structures of the pores and the mechanisms of the physical and chemical processes that occur during the activation process. It should be stressed that only a simultaneous analysis of the adsorption isotherms using the LBET and QSDFT methods makes it possible to choose the optimal preparation conditions considering the properties of the original raw material needed to obtain activated carbons dedicated to a particular purpose. However, the limitations of these methods must be accounted for, such as the assumption of a simplified model pore shape or simplifications of the process mechanisms, which often diverge from reality.

## Figures and Tables

**Table 1:** The microporous structure of activated carbons based on nitrogen adsorption isotherms, determined using the Brunauer–Emmett–Teller (BET), Dubinin-Radushkevitch (DR), and numerical clustering-based adsorption analysis (LBET) methods.

R	*S_BET_* m^2^/g	*V_DR_* cm^3^/g	*V_Total_* cm^3^/g	LBET No.	*V_hA_* cm^3^/g	*Q_A_*/*RT*	*B_C_*	*Z_A_*	*h*	*α*	*β*	*σ_e_*	*w_id_*
**The activated carbons prepared from mahogany via chemical activation with KOH (MAC/KOH)**													
1	896	0.451	0.498	4	0.418	−12.27	6.91	0.522	3	0.41	1.52	0.053	0.22
2	1697	0.673	0.760	10	0.639	−13.32	7.24	0.550	7	0.61	1.01	0.11	0.36
3	2496	0.935	1.210	2	1.425	−13.74	1.00	0.561	1	0.24	1.00	0.36	0.29
**The activated carbons prepared from ebony via chemical activation with KOH (EAC/KOH)**													
1	1121	0.496	0.521	22	0.462	−10.99	5.27	0.489	5	0.33	1.00	0.052	0.36
2	1938	0.796	0.885	28	0.777	−11.33	5.81	0.498	9	0.46	1.00	0.26	0.29
3	2735	0.918	1.298	2	1.514	−10.91	5.23	0.489	1	0.02	1.00	0.83	0.30
**The activated carbons prepared from hornbeam via chemical activation with KOH (HAC/KOH)**													
1	1009	0.434	0.478	7	0.401	−10.66	5.13	0.480	5	0.27	1.89	0.75	0.33
2	1770	0.754	0.838	19	0.743	−9.22	4.98	0.442	3	0.39	1.00	0.21	0.52
3	2795	0.937	1.281	2	1.454	−12.38	1.03	0.525	1	0.009	1.00	0.84	0.30
